# Supplementary material for: Polyketide synthase gene clusters in the ericoid mycorrhizal fungus Oidiodendron maius: genome-wide prediction, expression profiling, and functional characterization of knockout mutants
Source: Front Plant Sci. 2026 Sep 16;17:1924430. doi: 10.3389/fpls.2026.1924430 (PMC13623903; doi:10.3389/fpls.2026.1924430)
Supplement: Supplementary file 1 [file SupplementaryFile1.zip › Supplementary Materials/Supplementary Folder 1.zip!/Oidma1_AssemblyScaffolds_Repeatmasked/knownclusterblast/cluster17/jgi.p_Oidma1_119824_mibig_hits.html]

| MIBiG Protein | Description | MIBiG Cluster | MiBiG Product | % ID | % Coverage | BLAST Score | E-value |
| --- | --- | --- | --- | --- | --- | --- | --- |
| AAZ95013.1 | putative\_flavin-binding\_monooxygenase | BGC0000048 | Dothistromin\_biosynthetic\_gene\_cluster | 34 | 96.974789916 | 363 | 3.9e-100 |
| BAU98044.1 | Bayer\_Billiger\_monooxygenase | BGC0001386 | Isofuranonaphthoquinone\_biosynthetic\_gene\_cluster | 38 | 34.6218487395 | 161 | 3.9e-39 |
| ADC45584.1 | monooxygenase | BGC0000093 | Meilingmycin\_biosynthetic\_gene\_cluster | 24 | 77.9831932773 | 126 | 1.1e-28 |
| AFV30241.1 | fumarate\_reductase/succinate\_dehydrogenase\_flavoprotein\_domain\_protein | BGC0000075 | Hygrocin\_biosynthetic\_gene\_cluster | 30 | 36.4705882353 | 106 | 1.1e-22 |
| CBF80421.1 | conserved\_hypothetical\_protein | BGC0000680 | Austinol/dehydroaustinol,\_locus\_1\_biosynthetic\_gene\_cluster | 28 | 41.1764705882 | 105 | 1.9e-22 |
| BAC70705.1 | putative\_monooxygenase | BGC0000678 | Pentalenolactone\_biosynthetic\_gene\_cluster | 29 | 36.1344537815 | 102 | 1.6e-21 |
